# The assessment and management of patients with type 2 myocardial infarction: an international Delphi study

**DOI:** 10.1093/ehjqcco/qcaf069

**Published:** 2025-09-04

**Authors:** Caelan Taggart, Amy V Ferry, Andrew R Chapman, Stacey D Schulberg, Anda Bularga, Ryan Wereski, Jasper Boeddinghaus, Dorien M Kimenai, Matthew T H Lowry, Derek P Chew, Louise Cullen, Lori B Daniels, P J Devereaux, John French, Hanna K Gaggin, Thao Huynh, Laurent Jacquin, Allan S Jaffe, Tomas Jernberg, Ran Koronowski, Cian McCarthy, James McCord, Mamas A Mamas, Hans Mickley, David A Morrow, Christian Mueller, L Kristin Newby, William Parsonage, Claire E Raphael, Aiman Smer, Stephen W Smith, Yader Sandoval, Nathaniel R Smilowitz, Harvey White, Kai M Eggers, Bertil Lindahl, Kristian Thygesen, Nicholas L Mills

**Affiliations:** BHF Centre for Cardiovascular Science, University of Edinburgh, Chancellor’s Building, Edinburgh EH16 4SU, United Kingdom; BHF Centre for Cardiovascular Science, University of Edinburgh, Chancellor’s Building, Edinburgh EH16 4SU, United Kingdom; BHF Centre for Cardiovascular Science, University of Edinburgh, Chancellor’s Building, Edinburgh EH16 4SU, United Kingdom; BHF Centre for Cardiovascular Science, University of Edinburgh, Chancellor’s Building, Edinburgh EH16 4SU, United Kingdom; BHF Centre for Cardiovascular Science, University of Edinburgh, Chancellor’s Building, Edinburgh EH16 4SU, United Kingdom; BHF Centre for Cardiovascular Science, University of Edinburgh, Chancellor’s Building, Edinburgh EH16 4SU, United Kingdom; BHF Centre for Cardiovascular Science, University of Edinburgh, Chancellor’s Building, Edinburgh EH16 4SU, United Kingdom; BHF Centre for Cardiovascular Science, University of Edinburgh, Chancellor’s Building, Edinburgh EH16 4SU, United Kingdom; BHF Centre for Cardiovascular Science, University of Edinburgh, Chancellor’s Building, Edinburgh EH16 4SU, United Kingdom; Victorian Heart Hospital/Victorian Heart Institute, Monash University, Melbourne, VIC 3168, Australia; Faculty of Medicine, The University of Queensland, Brisbane, QLD 4072, Australia; Department of Medicine, University of California, San Diego, CA 92093, USA; Departments of Health Research Methods, Evidence, and Impact and Medicine, McMaster University, Hamilton, Canada L8S 4L8; Department of Cardiology, University of New South Wales and Liverpool Hospital, NSW 2170, Australia; Division of Cardiology, Department of Medicine, Massachusetts General Hospital, Harvard Medical School, Boston, MA 02114, USA; McGill University Health Centre, Montreal, Quebec, Canada H3G 1A4; Research Institute of McGill University Health Centre, Montreal, Quebec, Canada H3H 2R9; Emergency Medicine Department, Hospices Civils de Lyon, Edouard Herriot Hospital, Lyon 69003, France; CarMeN INSERM U1060, Lyon-1 University, Lyon 69310, France; Department of Cardiovascular Diseases, Mayo Clinic, Rochester, MN 55905, USA; Department of Laboratory Medicine and Pathology, Mayo Clinic, Rochester, MN 55905, USA; Department of Clinical Sciences, Danderyd Hospital, Karolinska Institutet, Stockholm 182 88, Sweden; Department of Cardiology, Rabin Medical Center, Petah Tikva, Faculty of Medicine, Tel Aviv University, Tel Aviv 49100, Israel; Division of Cardiology, Department of Medicine, Massachusetts General Hospital, Harvard Medical School, Boston, MA 02114, USA; Heart and Vascular Institute, Henry Ford Hospital, Detroit, MI 48307, USA; Keele Cardiovascular Research Group, Centre for Prognosis Research, Keele University, Keele ST5 5BG, United Kingdom; Department of Cardiology, Odense University Hospital, Odense 5000, Denmark; Cardiovascular Division, Department of Medicine, Brigham and Women's Hospital, Harvard Medical School, Boston, MA 02115, USA; Department of Cardiology and Cardiovascular Research Institute Basel (CRIB), University Hospital Basel, Basel 4031, Switzerland; Department of Medicine, Division of Cardiology, Duke Clinical Research Institute, Duke University Medical Center, Durham, NC 27705, USA; Australian Centre for Health Services Innovation, Queensland University of Technology, Brisbane, QLD 4059, Australia; Department of Cardiovascular Medicine, Mayo Clinic, Rochester, MN 55905, USA; CHI-Health-Creighton University School of Medicine, Omaha, NE 68131, USA; Department of Emergency Medicine, Hennepin County Medical Center and University of Minnesota, Minneapolis, MN 55415, USA; Minneapolis Heart Institute, Abbott Northwestern Hospital, Centre for Coronary Artery Disease, Minneapolis Heart Institute Foundation, Minneapolis, MN 55407, USA; Leon H. Charney Division of Cardiology, Department of Medicine, New York University Grossman School of Medicine, New York, NY 10016, USA; Te Toka Tumai, Green Lane Cardiovascular Services, Auckland City Hospital, Te Whatu Ora—Health, Auckland 1142, New Zealand; Department of Medical Sciences, Uppsala University, Uppsala 75123, Sweden; Department of Medical Sciences, Uppsala University, Uppsala 75123, Sweden; Department of Cardiology, Aarhus University Hospital, Aarhus 8200, Denmark; BHF Centre for Cardiovascular Science, University of Edinburgh, Chancellor’s Building, Edinburgh EH16 4SU, United Kingdom; Usher Institute, University of Edinburgh, Edinburgh EH16 4UX, United Kingdom

**Keywords:** Myocardial infarction, Type 2 myocardial infarction, Management, Consensus, Delphi study

## Abstract

**Aims:**

Type 2 myocardial infarction due to myocardial oxygen supply–demand imbalance is associated with poor outcomes. There are no guidelines to inform care for these patients. The consensus on the assessment and management of type 2 myocardial infarction is gained.

**Methods and results:**

An international e-Delphi study including experts in type 2 myocardial infarction identified through systematic review was conducted. Participants were asked to describe their approach to (i) definition and diagnosis, (ii) risk stratification, (iii) assessment of coronary artery disease and cardiac function, (iv) specialty management, (v) treatment and secondary prevention, and (vi) communication and rehabilitation. Statements generated in round one were circulated, with consensus defined *a priori* as ≥70% agreement on a 5-point Likert scale. Where no consensus was reached, statements were amended and recirculated for a final round. The response rate was 56% (38/68), 54% (37/68), and 72% (49/68) in the first, second, and third rounds, respectively. Following the first round, 67 unique statements were generated across six domains. Overall, consensus was achieved on 64% (43/67) of statements. Consensus was achieved for 42% (5/12) of statements on the diagnosis of type 2 myocardial infarction, 75% (3/4) on risk stratification, 50% (9/18) on the assessment of coronary artery disease and cardiac function, 60% (6/10), on specialty management, 100% (9/9) on treatment and secondary prevention, and 79% (11/15) on communication and rehabilitation.

**Conclusion:**

Consensus was obtained across a number of domains for the assessment and management of patients with type 2 myocardial infarction. However, there was limited agreement amongst experts on the diagnostic criteria, which may benefit from refinement.

## Graphical Abstract

**Graphical Abstract:**
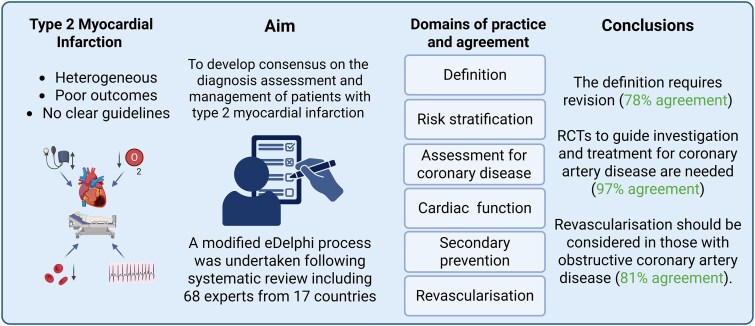

What is already known?

- Type 2 myocardial infarction is common, responsible for one in three myocardial infarction events in hospitalized patients over 70 years of age
- It is associated with poor short- and long-term outcomes, with an equivalent cardiovascular risk to type 1 myocardial infarction and just one in three patients alive at 5 years
- Despite poor outcomes, there is a lack of consensus on the optimal strategies for diagnosis, investigation and management of patients with type 2 myocardial infarction

What this study adds?

- Following systematic review, we conducted a Delphi study incorporating experts who have published in type 2 myocardial infarction
- We obtained consensus for recommendations across a number of domains including (i) definition and diagnosis, (ii) risk stratification, (iii) assessment of coronary artery disease and cardiac function, (iv) specialty management, (v) treatment and secondary prevention, and (vi) communication and rehabilitation.
- We believed this Delphi process will inform future discussion and the design of randomized controlled trials evaluating investigation and treatment strategies in type 2 myocardial infarction.

## Introduction

The Fourth Universal Definition of Myocardial Infarction defines five sub-types of myocardial infarction characterised by underlying aetiology.^1^ Type 1 myocardial infarction occurs due to atherosclerotic plaque rupture or erosion resulting in thrombus formation. In contrast, type 2 myocardial infarction occurs as a consequence of a reduction in myocardial oxygen supply or an increase in demand without evidence of acute atherothrombosis.

Type 2 myocardial infarction is common, responsible for between 7% and 62% of all myocardial infarction events depending on the clinical setting,^2-8^ and increasingly recognised due to the widespread adoption of high-sensitivity cardiac troponin assays.^2,9,10^ Two-thirds of patients with type 2 myocardial infarction are dead at five years, with cardiovascular outcomes comparable to patients type 1 myocardial infarction.^2,11,12^ Current guidelines do not differentiate management of myocardial infarction according to subtype.^13-15^ However, observational studies consistently demonstrate that patients with type 2 myocardial infarction are less likely to undergo investigation for coronary disease, coronary revascularization, or receive secondary preventation and cardiac rehabilitation compared to patients with type 1 myocardial infarction.^4,11,16-21^ Reflecting this uncertainty in practice, the American College of Cardiology and American Heart Association (AHA) have explicitly excluded type 2 myocardial infarction from myocardial infarction clinical performance and quality measures.^22^

Type 2 myocardial infarction encompasses both coronary and non-coronary mechanisms in a heterogeneous population.^2,23,24^ The classification is based on international consensus and whilst our knowledge of type 2 myocardial infarction is increasing^25-27^ no prospective randomized trials that have focused on type 2 myocardial infarction to guide care, and definitive evaluation is often not performed.^28,29^ Together, these issues have contributed to variation in the incidence and management of type 2 myocardial infarction across the world.^30,31^

We performed a systematic review to identify international experts in type 2 myocardial infarction and invited them to participate in an e-Delphi study with the aim of achieving consensus and informing strategies for the assessment and management of type 2 myocardial infarction.

## Methods

### Steering panel and oversight

A steering group was convened to oversee the study, which was approved by the Edinburgh University Research Ethics Committee (21-EMREC-030) and conducted in accordance with the Declaration of Helsinki. Information sheets were circulated to potential expert participants and written informed consent obtained. Data were anonymised at the point of collection (see Supplementary Material).

### Systematic review and participants

A systematic review of type 2 myocardial infarction was undertaken with search terms and databases as detailed in the supplement (see Supplementary material online, *Figure S1*). The initial screening of titles and abstracts was conducted by one investigator (CT), with full text review and agreement for inclusion obtained by consensus (CT, ARC and NLM). Of 424 articles identified, 114 reported original research on type 2 myocardial infarction (Supplementary Appendix including PRISMA checklist). All corresponding and lead authors were contacted and invited to participate. To improve the generalisability of our findings, we aimed to recruit experts from different regions across the world with broad representation from cardiology, internal medicine, and emergency medicine.

### Study process

**Figure 1:**
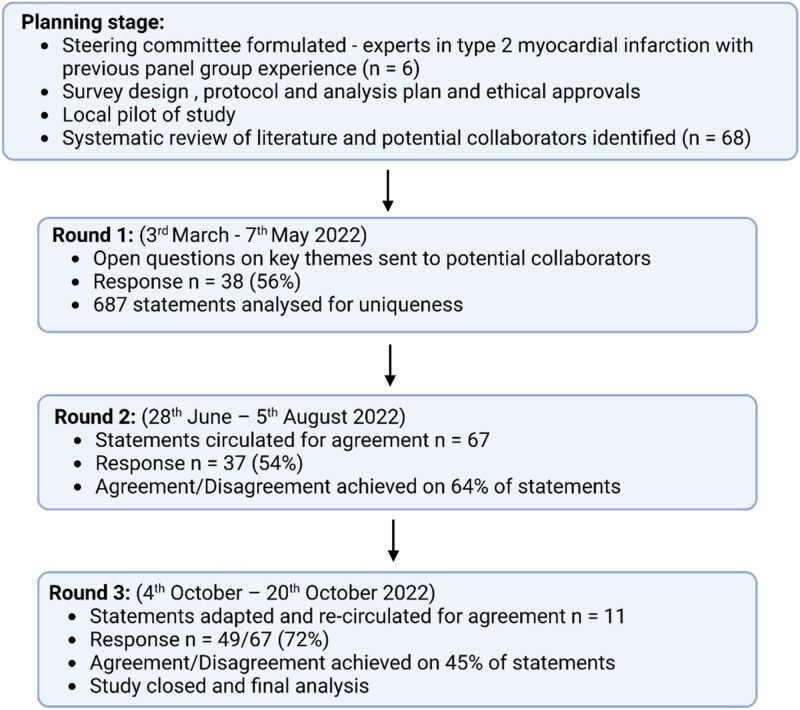
The delphi process.

Using standard methodology, an e-Delphi study was conducted in three rounds with established online survey tools (Jisc©, Bristol, UK) (*Figure 1*).^32-35^ The first round was exploratory and took place between 3 March and 7 May 2022. The steering committee posed a series of questions to participants to understand their approach to the care of patients with type 2 myocardial infarction across six domains: i) definition and diagnosis, ii) risk stratification, iii) assessment of coronary artery disease and cardiac function, iv) specialty management, v) treatment and secondary prevention, and vi) communication and rehabilitation (Supplementary Appendix). Statements generated from round one underwent deductive qualitative analysis using specialist software (NVivo © version 12.1, QSR International, Burlington Massachusetts, USA) and were grouped for uniqueness. The second round took place between 28 June and 5 August 2022. Unique statements were circulated with eligible participants asked to provide a level of agreement on a five-point Likert scale with the following criteria: (1) strongly agree, (2) agree, (3) neither agree or disagree, (4) disagree, or (5) strongly disagree. Statements were retained if recommendations were agreed, or were recirculated in a third round. Consistent with prior studies, consensus was defined *a priori* as ≥70% of participants agreeing (agree or strongly agree) or disagreeing (disagree or strongly disagree).^32-35^ Participants were provided with the opportunity to give written feedback which was available to the steering panel to inform the third round. This took place between 4 and 20 October 2022, and comprised statements where no consensus had been reached.

### Statistical analysis

Descriptive statistics were used to illustrate the distribution of responses for each statement in rounds two and three, with the level of agreement was reported as a median and interquartile range (IQR) for each statement from the Likert scale. Consensus was defined as the proportion of participants (%) agreeing (agree or strongly agree) or disagreeing (disagree or strongly disagree). All data analysis was performed in R Studio (version 3.6).

## Results

### Response rate and participant characteristics

The systematic review identified 73 potential participants from 19 countries across 4 continents. There was a 56% (38/68), 54% (37/68) and 72% (49/68) response rate in round one, two and three, respectively. The majority of participants worked in cardiology (84%; 32/38), with the remainder working in emergency medicine (13%; 5/38) and in internal medicine (3%, 1/38) (see Supplementary material online, *Table S1*).

### Rounds one and two

In round one, 15 broad questions were posed across six domains of practice (Supplementary Appendix). From the response, 687 individual statements were extracted, with deductive analysis grouping similar statements. The steering group identified 67 unique statements that were circulated in round two (see Supplementary material online, *Table S1*). Overall consensus was achieved on 64% (43/67) of statements.

(i) Definition and diagnosis of type 2 myocardial infarction

**Figure 2:**
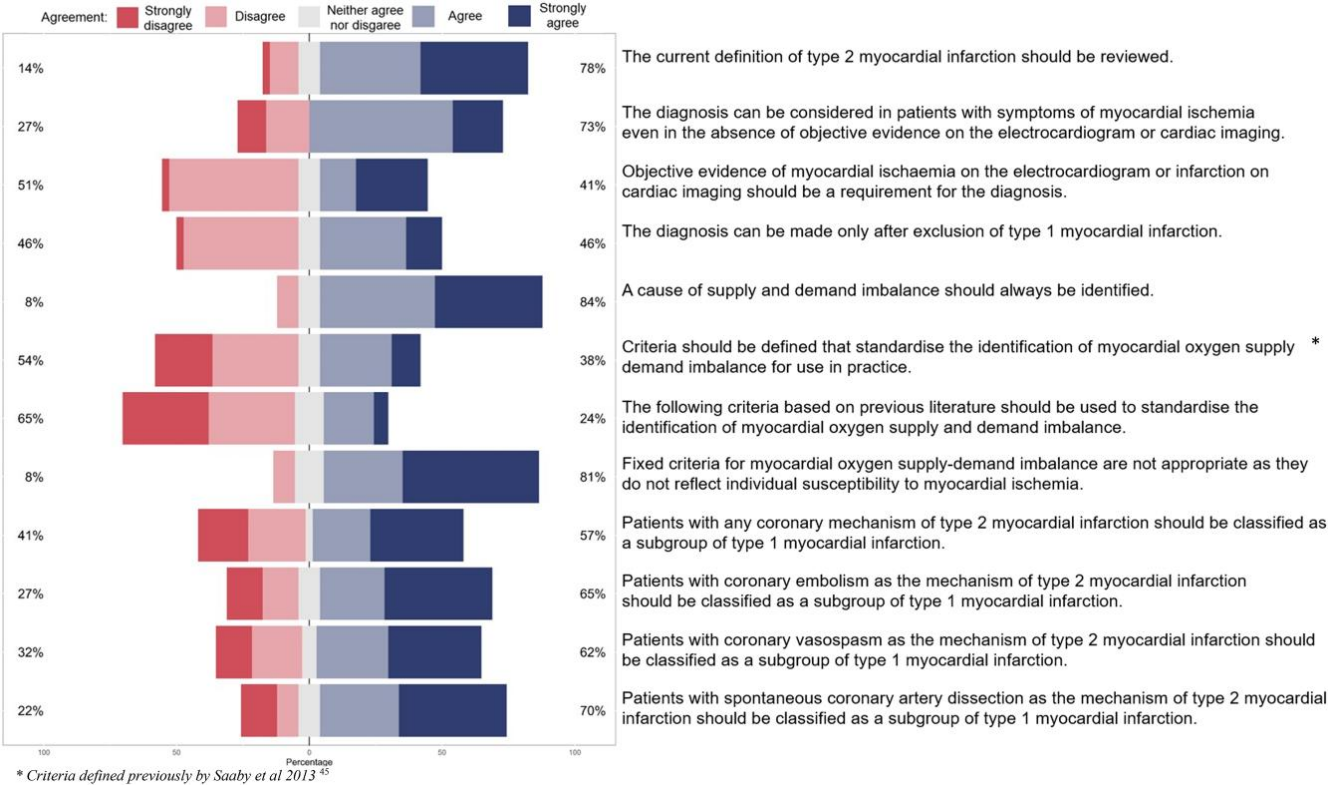
Statements and agreement on the definition and diagnosis of type 2 myocardial infarction.

Consensus was achieved on 42% (5/12) of statements (*Figure 2*). The majority of participants (78%; 29/37) agreed that the diagnostic criteria for type 2 myocardial infarction should be reviewed [median (IQR) 2 (1), lower numbers indicate greater agreement]. Consensus was also reached in 73% (27/37) of participants that the diagnosis of type 2 myocardial infarction could be considered in patients with symptoms, in the absence of objective evidence of myocardial ischaemia [median (IQR) 2 (2)]. Participants did not reach consensus on whether coronary embolism or coronary vasospasm should be reclassified as type 1 myocardial infarction; however, there was a consensus on reclassifying spontaneous coronary artery dissection as type 1 myocardial infarction in 70% (26/37); median (IQR) 2 (2; *Figure 2*).

Box 1 Statements on the definition and diagnosis of type 2 myocardial infarction where there was agreement (%) by consensus

**Table:** 

The current definition of type 2 myocardial infarction should be reviewed	78%
The diagnosis can be considered in patients with symptoms of myocardial ischaemia even in the absence of objective evidence on the electrocardiogram or cardiac imaging	73%
A cause of supply and demand imbalance should always be identified	84%
Fixed criteria for myocardial oxygen supply-demand imbalance are not appropriate as they do not reflect individual susceptibility to myocardial ischaemia	81%
Patients with spontaneous coronary artery dissection should be classified as a subgroup of type 1 myocardial infarction	70%

(ii) Risk stratification in patients with type 2 myocardial infarction

**Figure 3:**
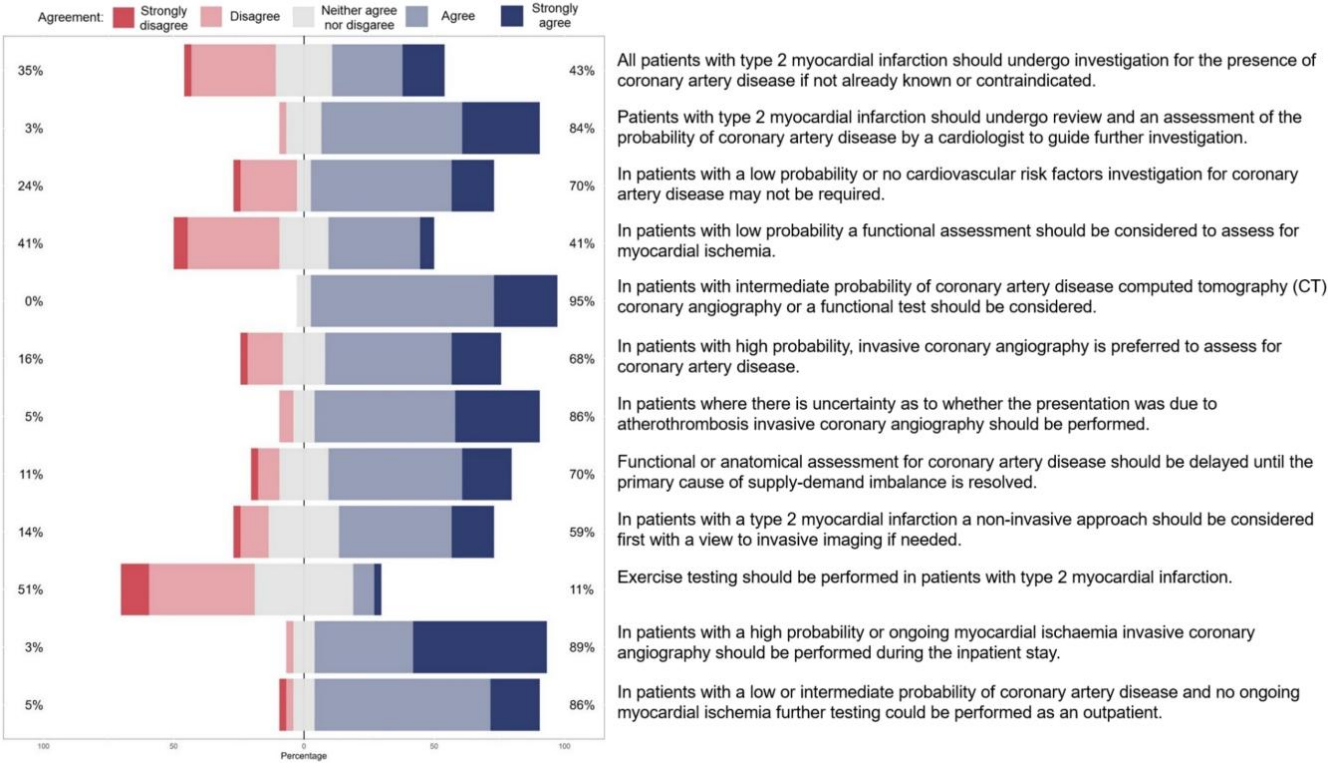
Statements and agreement on the assessment for coronary artery disease and cardiac function in type 2 myocardial infarction.

Consensus was achieved on 75% (3/4) of statements on risk stratification (see Supplementary material online, *Figure S3*). There was consensus amongst 73% (27/37); (median [IQR] 2 [1]) of participants that the same approach to risk stratification should be applied in patients with type 1 and type 2 myocardial infarction, and 97% (36/37); (median [IQR] 2 [1]) agreed and that this assessment should be pragmatic and include a clinical review to evaluate the probability of coronary artery disease and impaired cardiac function (*Figure 3*). The majority (92%, 34/37); (median [IQR] 1 [1]) also strongly agreed that new risk stratification tools are needed to help clinicians target cardiac investigations and treatments in patients with type 2 myocardial infarction.

Box 2 Statements on risk stratification for patients with type 2 myocardial infarction where there was agreement (%) by consensus

**Table:** 

Patients with type 2 myocardial infarction should be risk stratified in the same way as those with type 1 myocardial infarction	73%
Risk stratification should be pragmatic and include a clinical review to evaluate the probability of coronary artery disease and impaired cardiac function	97%
New risk stratification tools are needed to evaluate prognosis and support clinicians to target further cardiac investigation and treatment in type 2 myocardial infarction	92%

(iii) Assessment for coronary artery disease and cardiac function in patients with type 2 myocardial infarction

**Figure 4:**
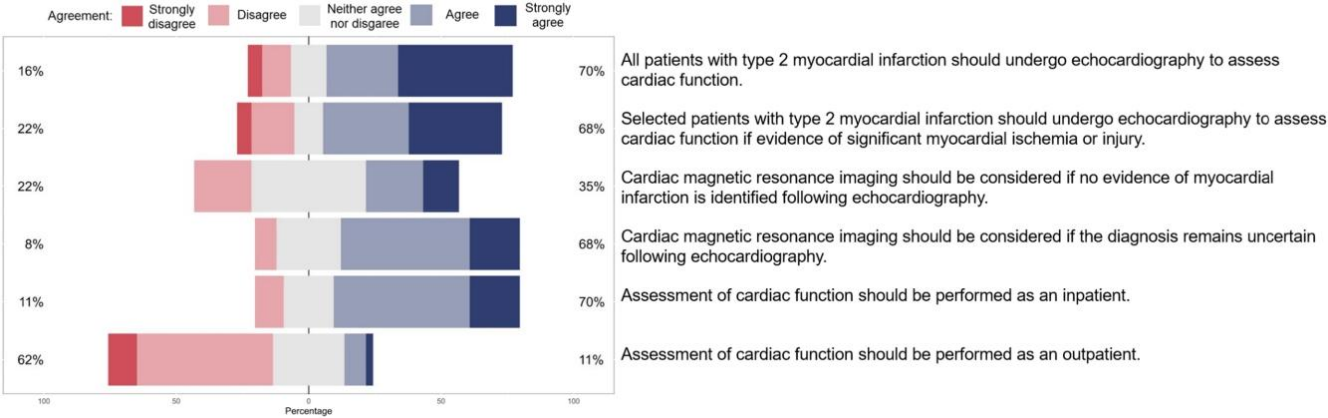
Statements and agreement on the assessment of cardiac function in type 2 myocardial infarction.

On the assessment of patients with type 2 myocardial infarction, consensus was achieved on 50% (9/18) of the statements (*Figures 3* and *4*). The majority agreed that patients should undergo review and assessment of the probability of coronary artery disease by a cardiologist to guide further investigation (84%) with further recommendations as outlined in Box 3.

Box 3 Statements on the assessment of patients with type 2 myocardial infarction where there was agreement (%) by consensus

**Table:** 

Patients with type 2 myocardial infarction should undergo review and an assessment of the probability of coronary artery disease by a cardiologist to guide further investigation	84%
In patients with a low probability or no cardiovascular risk factors, investigation for coronary artery disease may not be required	70%
In patients with intermediate probability of coronary artery disease computed tomography (CT) coronary angiography or a functional test should be considered	95%
In patients where there is uncertainty as to whether the presentation was due to atherothrombosis invasive coronary angiography should be performed	86%
Functional or anatomical assessment for coronary artery disease should be delayed until the primary cause of supply-demand imbalance is resolved	70%
In patients with a high probability or ongoing myocardial ischaemia invasive coronary angiography should be performed during the inpatient stay	89%
In patients with a low or intermediate probability of coronary artery disease and no ongoing myocardial ischaemia further testing could be performed as an outpatient	86%
All patients with type 2 myocardial infarction should undergo echocardiography to assess cardiac function	70%
Assessment of cardiac function should be performed as an inpatient	70%

(iv) Specialty management of patients with type 2 myocardial infarction

**Figure 5:**
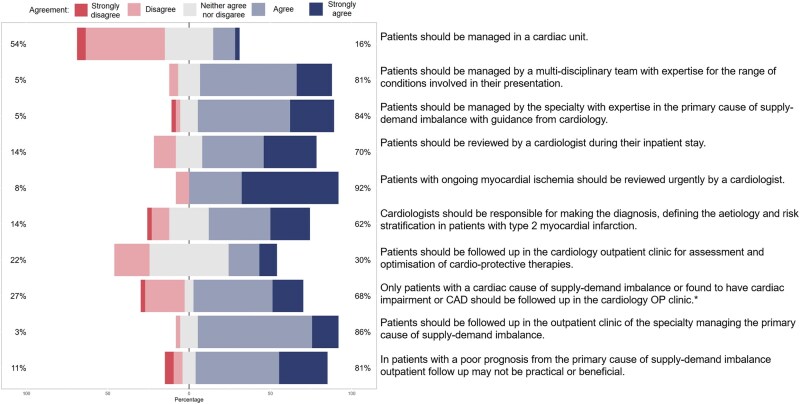
Statements and agreement on the specialty management of type 2 myocardial infarction.

On the role of specialty management of patients with type 2 myocardial infarction, consensus was achieved on 60% (6/10) of statements (*Figure 5*). There was a consensus across 81% (30/37); [median (IQR) 2 (1)] of participants that patients with type 2 myocardial infarction should be managed by a multi-disciplinary team. There was also a clear consensus that patients with type 2 myocardial infarction should be evaluated by a cardiologist during their inpatient stay, and strong agreement that this review should be conducted urgently in patients with ongoing myocardial ischaemia. The majority of participants agreed that outpatient follow-up should be arranged by the speciality responsible for the primary presenting condition, and that outpatient assessment by a cardiologist may not be practical or beneficial where the prognosis from the primary condition is poor.

Box 4 Statements on the management of patients with type 2 myocardial infarction where there was agreement (%) by consensus

**Table:** 

Patients should be managed by a multi-disciplinary team with expertise for the range of conditions involved in their presentation	81%
Patients should be managed by the specialty with expertise in the primary cause of supply–demand imbalance with guidance from cardiology	84%
Patients should be reviewed by a cardiologist during their inpatient stay	70%
Patients with ongoing myocardial ischaemia should be reviewed urgently by a cardiologist	92%
Patients should be followed up in the outpatient clinic of the specialty managing the primary cause of supply-demand imbalance	86%
In patients with a poor prognosis from the primary cause of supply-demand imbalance outpatient follow up may not be practical or beneficial	81%

(v) Treatment and secondary prevention in patients with type 2 myocardial infarction

Consensus was achieved on all nine unique statements regarding the treatment and use of secondary prevention (see Supplementary material online, *Figure S4*). Ninety-seven percent (36/37) of participants agreed or strongly agreed that management should include optimisation of treatment for the underlying condition causing supply demand imbalance to prevent recurrent type 2 myocardial infarction. Most participants strongly agreed that preventative therapies should be initiated in patients identified as having coronary artery disease or cardiac impairment or in those considered to be at intermediate or high risk of future cardiovascular events but should not be initiated in all patients with type 2 myocardial infarction. It was agreed that coronary revascularization should be considered in patients with obstructive coronary artery disease who are likely to have recurrent symptoms on effort or with further episodes of supply demand imbalance, despite optimal medical therapy or in those with left main stem disease or multivessel disease and cardiac impairment, as it may confer prognostic benefit.

Box 5 Statements on the treatment of patients with type 2 myocardial infarction where there was agreement (%) by consensus

**Table:** 

Revascularization should be considered in patients with obstructive coronary artery disease who are likely to have recurrent symptoms of myocardial ischaemia on effort or with further episodes of supply–demand imbalance despite optimal medical therapy	81%
Revascularization should be considered in patients with left main stem disease or multivessel disease and cardiac impairment as it may confer prognostic benefit	97%
Preventative therapies, such as aspirin and lipid-lowering therapy, should be initiated in patients identified with coronary artery disease if no contraindications?	95%
Preventative therapies, such as aspirin and lipid-lowering therapy, should be initiated in identified with coronary artery disease or at intermediate or high risk of cardiovascular events if no contraindications?	92%
Preventative therapies, such as angiotensin-converting enzyme (ACE) inhibitors, angiotensin receptor blockers, and beta-blockers, should be initiated in patients identified with cardiac impairment if no contraindications	86%
Preventative therapies, such as ACE inhibitors, angiotensin receptor blockers, and beta-blockers, should be initiated in patients identified with cardiac impairment or at intermediate or high risk of cardiovascular events if no contraindications	73%
Management should include optimization of treatment for the underlying condition causing supply–demand imbalance to prevent recurrent type 2 myocardial infarction	97%
Statements on the treatment of patients with type 2 myocardial infarction where there was disagreement (%) by consensus	
Preventative therapies, such as aspirin and statins, should be initiated in all patients with type 2 myocardial infarction if no contraindications	70%
Preventative therapies, such as ACE inhibitors, angiotensin receptor blockers, and beta-blockers, should be initiated in all patients with type 2 myocardial infarction if no contraindications	70%

(vi) Communication and rehabilitation in patients with type 2 myocardial infarction

On the importance of communication and cardiac rehabilitation in patients with type 2 myocardial infarction, consensus was achieved on 79% (11/15) of statements (see Supplementary material online, *Figure S5*). Ninety-seven percent (36/37) of participants disagreed with the statement that patients and clinicians have a good understanding of type 2 myocardial infarction. There was agreement in all participants that the explanation of the diagnosis should emphasise the underlying condition causing supply demand imbalance, and that the importance of managing cardiovascular risk factors should be communicated to patients. A majority (76%) of participants agreed that patients with type 2 myocardial infarction should be informed they are at increased risk of future myocardial infarction or death. However, there was no consensus on whether use of the term heart attack is appropriate when explaining the diagnosis of type 2 myocardial infarction to patients and relatives (see Supplementary material online, *Figure S5*).

Box 6 Statements on communication and rehabilitation of patients with type 2 myocardial infarction where there was agreement (%) by consensus

**Table:** 

Explanation of the diagnosis of type 2 myocardial infarction should be described in language understandable to the patient	81%
When the diagnosis is explained to patients’ extra consideration should be given to the condition causing supply-demand imbalance and the importance of preventing this	100%
The importance of managing cardiovascular risk factors should be communicated to the patient	100%
That patients with type 2 myocardial infarction are at increased risk of having a future heart attack or dying should be communicated to the patient	76%
Cardiac rehabilitation should be offered on a case-by-case basis. There may be limited benefit in very young and very elderly patients.	70%
This should include lifestyle and dietary advice as for type 1 myocardial infarction	81%
Patient information leaflets and online information should be developed within health systems to improve patient understanding of type 2 myocardial infarction	89%
Educational resources are needed for clinicians to improve understanding of type 2 myocardial infarction	95%
Randomised trials of investigations and treatments for coronary artery disease and cardiac impairment are needed to improve outcomes in type 2 myocardial infarction	97%
Statements on communication and rehabilitation of patients with type 2 myocardial infarction where there was disagreement (%) by consensus	
Patients have a good understanding of the diagnosis of type 2 myocardial infarction	97%
Clinicians have a good understanding of the diagnosis of type 2 myocardial infarction	92%

Additional comments from the participants were often insightful and selected comments are included in the Supplementary material online, Supplementary Appendix.

### Round three

Consensus was not achieved on 36% (24/67) of the unique statements circulated in round two, which were reviewed by the steering group and reformatted as 11 unique statements for round 3 (see Supplementary material online, *Table S2*). No consensus was reached on 55% (6/11) of these statements, including if objective evidence of myocardial ischaemia on the electrocardiogram or new evidence of infarction on cardiac imaging should be a requirement for the diagnosis of type 2 myocardial infarction. Likewise, there was no consensus on whether the diagnosis should be restricted to those with a presumed or demonstrable coronary mechanism or on further subclassification of type 2 myocardial infarction into those with (type 2a) or without (type 2b) a coronary mechanism. There was consensus that cardiac magnetic resonance imaging should be considered in those where the diagnosis remained uncertain following echocardiography and coronary angiography. Interestingly, although there was no consensus on restricting the diagnosis to those with coronary mechanisms or for use of the term ‘heart attack’ in all patients with type 2 myocardial infarction, 88% (43/49) and 84% (41/49) agreed the term ‘heart attack’ was appropriate when explaining the diagnosis to patients and relatives where infarction was caused by a coronary mechanism or resulted in new evidence of infarction on cardiac imaging, respectively (see Supplementary material online, *Figure S2*, Supplementary material online, *Table S3*).

Box 7 Statements in round three there was agreement (%) by consensus

**Table:** 

In patients with a high probability of coronary artery disease in whom further investigation is appropriate, invasive coronary angiography is preferred	73%
Cardiac magnetic resonance imaging should be considered if the diagnosis remains uncertain following echocardiography and/or coronary angiography	88%
Patients with a cardiac cause of supply-demand imbalance or found to have cardiac impairment or coronary artery disease should be followed up in the cardiology outpatient clinic	98%
Use of the term heart attack is appropriate when explaining the diagnosis of type 2 myocardial infarction to patients and relatives where infarction was caused by a coronary mechanism	88%
Use of the term heart attack is appropriate when explaining the diagnosis of type 2 myocardial infarction to patients and relatives where there is new evidence of myocardial infarction on cardiac imaging	84%

## Discussion

We conducted an international Delphi study to document and understand expert opinion on the assessment and management of patients with type 2 myocardial infarction and aimed to identify areas where there is a consensus to inform practice and guide future research.

There remains uncertainty around the optimal definition of type 2 myocardial infarction. At present, subjective symptoms and objective signs of ischaemia are given equal weighting in the definition, even though there is recognition that the latter are more frequently associated with abnormalities on cardiac imaging and adverse prognosis.^29^ We observed consensus agreement in upholding the current approach; however, this lack of objective criteria may continue to pose challenges if multi-centre clinical trials are undertaken in patients with type 2 myocardial infarction, due to heterogeneity in the interpretation of cardiac symptoms. Furthermore, differentiating type 2 myocardial infarction from acute non-ischaemic myocardial injury is challenging and there may be clinical diagnostic overlap and misclassification in practice.^2,36^ The definition of type 2 myocardial infarction encompasses a variety of coronary and non-coronary pathologies that have little in common.^1^ Although patients with coronary vasospasm, coronary embolism, and spontaneous coronary dissection often present with ST-segment elevation and are initially managed and triaged in the same way as patients with type 1 myocardial infarction, following diagnosis, there are clear differences in recommendations for patient care. Whilst there was consensus that patients with spontaneous coronary artery dissection should be reclassified as type 1 myocardial infarction, there was no consensus on whether other coronary phenotypes should be reclassified. It is important to emphasize that these presentations are less common, and their relative prevalence is low.^23^ A diagnostic framework in which patients presenting with acute coronary pathology are more closely aligned, but the underlying coronary mechanisms are clearly defined may be more intuitive.^37^

Current approaches to risk stratification are hindered by a lack of consensus around the relative importance of traditional cardiovascular risk factors,^38^ and whether outcomes simply reflect patient age or non-modifiable comorbidities. There are, to date, no intervention trials that have focussed on type 2 myocardial infarction. The Delphi process indicated that traditional risk stratification approaches used in patients with type 1 myocardial infarction could be applied in type 2 myocardial infarction, but that new tools were needed to help target investigation and treatment. The value of using traditional risk stratification tools like GRACE 2.0 is unknown, and this tool has been shown to have only moderate discrimination for prediction all cause death.^39^ Bespoke tools may provide alternatives to conventional risk stratification models, with the recently derived T2-Risk score demonstrating improved performance over GRACE 2.0 for the prediction of myocardial infarction or all cause death.^40,41^

Optimal strategies for the use of cardiac investigations and treatments in type 2 myocardial infarction have not been defined. Previous efforts to understand the mechanism of myocardial injury have identified a high burden of unrecognized and untreated coronary and structural heart disease.^23,42^ The Delphi process reached consensus that a multi-disciplinary team should provide recommendations for optimal care for patients with type 2 myocardial infarction, with input from a cardiologist to guide investigation based on the likelihood of underlying coronary or structural heart disease. In the absence of ongoing ischaemia or a high probability of coronary artery disease, a non-invasive approach in the outpatient setting was considered appropriate with computed tomography coronary angiography or functional testing, and recommendations for secondary prevention therapy in line with guideline directed optimal medical therapy where appropriate.

There is no evidence to support routine coronary angiography or revascularization in patients with type 2 myocardial infarction, as trials comparing early invasive and conservative approaches predated the universal definition. However, observational studies have demonstrated patients with type 2 myocardial infarction are at increased risk of future type 1 myocardial infarction and recurrent type 2 myocardial infarction.^2,38^ Therefore, where obstructive coronary disease is identified and symptoms of angina are present, the Delphi participants agreed revascularization may be considered as it might reduce risk of recurrent symptoms or confer prognostic benefit. There is a tension between risk of invasive investigation and potential benefit in an older population with comorbid illness and increased bleeding risk at increased risk of complications, and clearly this risk/benefit assessment requires evaluation in clinical trials.

Nearly all participants agreed that patients and clinicians have a poor understanding about type 2 myocardial infarction. Clearly, there is an unmet need for educational resources, which should be developed in conjunction with patients who have experienced this condition. In nearly all domains, there was consensus that further research and randomized controlled trials were required.^42-44^ For trials to be successfully delivered in patients with type 2 myocardial infarction, designs will need to be pragmatic, with minimal exclusion criteria, and trial infrastructure will need to recruit across multiple centres. In this heterogenous population, a single intervention is unlikely to be effective and complex or even patient-specific interventions may be more appropriate. This heterogeneity may lend itself to adaptive clinical trial design with enrichment for clinical phenotypes, for example, by targeting revascularization or anti-ischaemic pharmacotherapy to patients with or without obstructive coronary artery disease to modify ischaemic substrate, or to target antiplatelet or statin therapy to all with coronary artery disease to reduce future cardiovascular risk.

### Limitations

Our systematic review identified participants with expertise in type 2 myocardial infarction across many different healthcare systems improving generalizability. We applied robust Delphi methodology allowing our full panel to suggest recommendations for further refinement. Responses were submitted anonymously, and participants were blinded to each other’s responses. However, we relied on prior peer reviewed publication to identify experts with understanding of type 2 myocardial infarction; therefore, clinicians with a particular interest or those from other specialties who encounter this condition more frequently may not have been identified. Our systematic review identified a lower proportion of female experts, and no participants from Africa or South America. This may affect the generalizability of our findings as lower income countries may not have access to the level of imaging and assessment modalities, which were suggested by expert consensus. Due to data protection concerns, which were raised during ethical review, we did not collect detailed information on participants place of work or whether this was at a district general or tertiary centre to reduce the likelihood of identification. Furthermore, we had a variable response rate across the three rounds with only 56% and 54% of possible participants contributing to round one and round two, respectively. Although consensus was reached across a number of domains, investigation and treatment recommendations are expert opinion and not based on randomized controlled trials. Therefore, clinicians must continue to approach the risk stratification, investigation, and treatment of type 2 myocardial infarction on an individual patient basis.

## Conclusions

Whilst considerable uncertainty remains, an international e-Delphi study has obtained consensus across several domains for the assessment and management of patients with type 2 myocardial infarction. Further research is needed to evaluate these approaches and provide an evidence base to guide care in clinical practice.

## Supplementary Material

qcaf069_Supplementary_Data

## Data Availability

The anonymized data underlying this article are available in the article and in its online supplementary material.
