# Supplementary material for: The assessment and management of patients with type 2 myocardial infarction: an international Delphi study
Source: Eur Heart J Qual Care Clin Outcomes. 2025 Sep 4;11(8):1421–30. doi: 10.1093/ehjqcco/qcaf069 (PMC12714377; doi:10.1093/ehjqcco/qcaf069)
Supplement: qcaf069_Supplementary_Data [file qcaf069_supplementary_data.docx]

SUPPLEMENTARY MATERIAL

**The assessment and management of patients with type 2 myocardial infarction: an international Delphi study**

**Running title:** Delphi study of type 2 myocardial infarction

Caelan Taggart MD^1*^, Amy V Ferry PhD^1*^, Andrew R Chapman MD, PhD^1*^,

Stacey D Schulberg MSc^1^, Anda Bularga MD^1^, Ryan Wereski MD^1^,

Jasper Boeddinghaus MD^1^, Dorien M Kimenai PhD^1^ , Matthew TH Lowry MD^1^,

Derek P. Chew MD, MPH, PhD^2^, Louise Cullen MD, PhD^3^, Lori B. Daniels MD^4^,

PJ Devereaux MD, PhD^5^, John French MD, PhD^6^, Hanna K. Gaggin MD, MPH^7^,

Thao Huynh MD PhD^8^, Laurent Jacquin MD, PhD^9^, Allan S. Jaffe MD ^10^,

Tomas Jernberg MD, PhD^11^, Ran Koronowski MD^12^, Cian McCarthy, MD, SM^7^,

James McCord MD, PhD^13^, Mamas A. Mamas MD, DPhil^14^, Hans Mickley MD, DMSc^15^,

David A. Morrow MD, MPH^16^, Christian Mueller, MD^17^, L. Kristin Newby MD, MHS^18^,

William Parsonage MD, DM^19^, Claire E. Raphael MD, PhD^20^, Aiman Smer MD^21^,

Stephen W. Smith MD, PhD^22^, Yader Sandoval MD^23^, Nathaniel R. Smilowitz MD, MS^24^ Harvey White MD PhD^25^, Kai M Eggers MD, PhD^26^, Bertil Lindahl MD, PhD^26^,

Kristian Thygesen MD, PhD^27^, Nicholas L Mills MD, PhD^1,28^

*^1^ BHF Centre for Cardiovascular Science, University of Edinburgh, Edinburgh, United Kingdom*

*^2^* *Victorian Heart Hospital/Victorian Heart Institute, Monash University, Melbourne, Australia*

*^3^ Faculty of Medicine, The University of Queensland, Brisbane, Australia*

*^4^ Department of Medicine, University of California, San Diego, USA*

*^5^ Departments of Health Research Methods, Evidence, and Impact and Medicine, McMaster University, Hamilton, Canada*

*^6^ Department of Cardiology, University of New South Wales & Liverpool Hospital, New South Wales, Australia
^7^ Division of Cardiology, Department of Medicine, Massachusetts General Hospital, Harvard Medical School, Boston, Massachusetts, USA*

*^8^ McGill University Health Centre, Montreal, Quebec, Canada; Research Institute of McGill University Health Centre, Montreal, Quebec, Canada*

*^9^ Emergency medicine department ,Hospices Civils de Lyon, Edouard Herriot Hospital, Lyon, France; CarMeN INSERM U1060, Lyon-1 university, Lyon, France*

*^10^ Department of Cardiovascular Diseases, Mayo Clinic, Rochester, Minnesota and department of Laboratory Medicine and Pathology, Mayo Clinic, Rochester, Minnesota, USA*

*^11^ Department of clinical sciences, Danderyd hospital, Karolinska Institutet, Stockholm, Sweden*

*^12^ Department of Cardiology, Rabin Medical Center, Petah Tikva, Faculty of Medicine, Tel Aviv University, Tel Aviv, Israel*

*^13^ Heart and Vascular Institute, Henry Ford Hospital, Detroit, Michigan, USA*

*^14^ Keele Cardiovascular Research Group, Centre for Prognosis Research, Keele University, Keele, United Kingdom*

*^15^ Department of Cardiology, Odense University Hospital, Odense, Denmark*

*^16^ Cardiovascular Division, Department of Medicine, Brigham and Women's Hospital, Harvard Medical School, Boston, Massachusetts, USA*

*^17^ Department of Cardiology and Cardiovascular Research Institute Basel (CRIB), University Hospital Basel, Basel, Switzerland*

*^18^ Department of Medicine, Division of Cardiology, Duke Clinical Research Institute, Duke University Medical Center, Durham, North Carolina, USA*

*^19^ Australian Centre for Health Services Innovation, Queensland University of Technology, Queensland, Australia*

*^20^ Department of Cardiovascular Medicine, Mayo Clinic, Rochester, Minnesota, USA*

*^21^ CHI-Health-Creighton University School of Medicine, Omaha, Nebraska, USA*

*^22^ Department of Emergency Medicine, Hennepin County Medical Center and University of Minnesota, Minneapolis, Minnesota, USA*

*^23^ Minneapolis Heart Institute, Abbott Northwestern Hospital, Centre for Coronary Artery Disease, Minneapolis Heart Institute Foundation, Minneapolis, Minnesota, USA*

*^24^ Leon H. Charney Division of Cardiology, Department of Medicine, New York University Grossman School of Medicine, New York, USA*

*^25^ Te Toka Tumai, Green Lane Cardiovascular Services, Auckland City Hospital, Te Whatu Ora – Health New Zealand, Auckland, New Zealand*

*^26^ Department of Medical Sciences, Uppsala University, Uppsala, Sweden*

*^27^ Department of Cardiology, Aarhus University Hospital, Aarhus, Denmark*

*^28^ Usher Institute, University of Edinburgh, Edinburgh, United Kingdom*

**These authors contributed equally*

**Corresponding author:**

Nicholas L Mills, MD, PhD

Centre for Cardiovascular Science

The University of Edinburgh

Chancellor’s Building

Edinburgh EH16 4SU

United Kingdom

Email: [nick.mills@ed.ac.uk](mailto:nick.mills@ed.ac.uk)

Twitter: @HighSTEACS

**Ethical review**

The study was conducted with approval from the Edinburgh Medical School Research Ethics Committee EMREC, Study number 21-EMREC-030. All potential and consenting participants to this study had their responses anonymised to each other and to the reviewers and steering group. A full information summary was circulated to potential participants so they could make an informed choice on whether they wished to take part. All potential participants had the option of having their name removed from the emailing list. Written consent was gained at the beginning of each Delphi round of questioning. Upon the completion of all three rounds of the Delphi process, participants were offered the opportunity to contribute to the original research article.

**Systematic review process – As per PRIMSA**

Indication: A systematic review was performed to identify experts in type 2 myocardial infarction.

Eligibility criteria: Search terms included “type 2 myocardial infarction”, “type two myocardial infarction” or “Universal definition of myocardial infarction”. This search was restricted to original research articles in English and did not include editorials, commentaries or case reports.

Information sources: <https://pubmed.ncbi.nlm.nih.gov/> was searched with the terms highlighted in the eligibility criteria between dates from January 1^st^ 2007 and September 27^th^ 2021. These dates where chosen as they reflect the first definition of type 2 myocardial infarction in 2007.

Risk of bias: As the systematic review was undertaken to identify those who have published in this area, no formal risk of bias assessment was undertaken.

Synthesis of results: Data of each article was collated with authors for each article defined.

Included studies: 114 articles were included.

Synthesis of results: N/A

Limitations of evidence: N/A

Interpretation: No synthesis or analysis was undertaken using the systematic review.

Funding: This review was funded as part of the research grant by the British Heart Foundation (FS/CRTF/21/2473)

**Supplementary Figure 1.** Systemic search methodology flow diagram to identify potential collaborators for the study

**
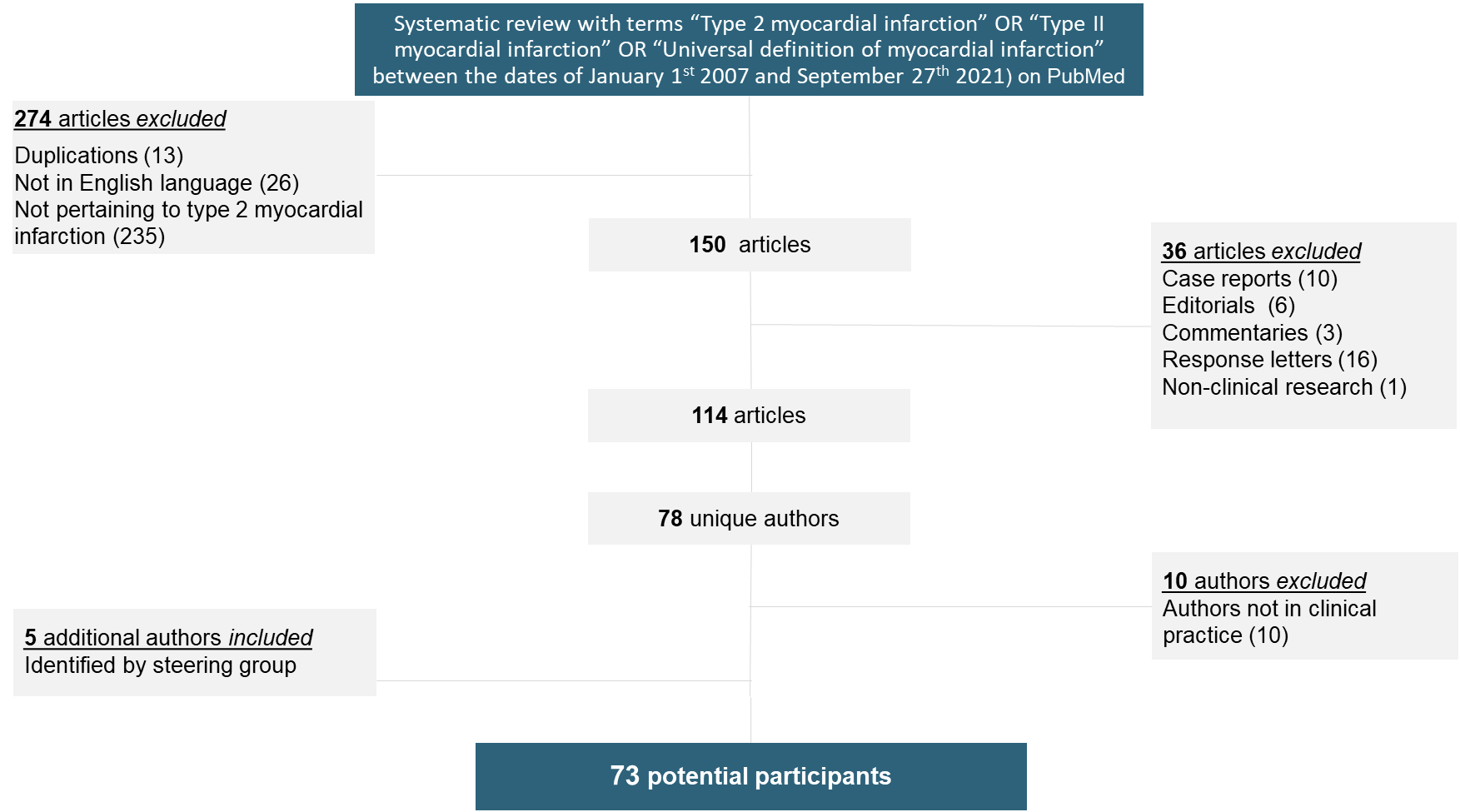
**

Search for terms “Type 2 myocardial infarction” OR “Type II myocardial infarction” OR “Universal definition of myocardial infarction” between the dates of January 1^st^ 2007 and September 27^th^ 2021 on <https://pubmed.ncbi.nlm.nih.gov/> identified 424 publications. Articles where removed as above and corresponding or lead authors identified.


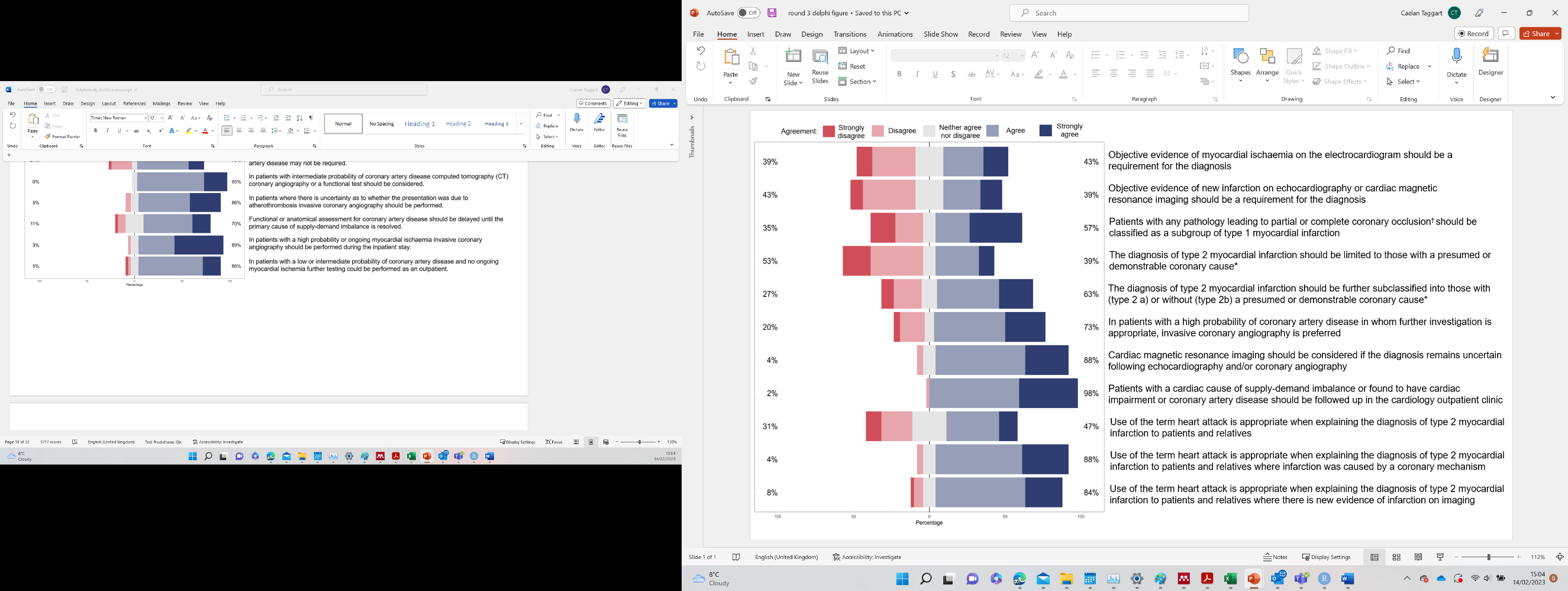
**Supplementary Figure 2:** Round three statements re-circulated for consensus.

**
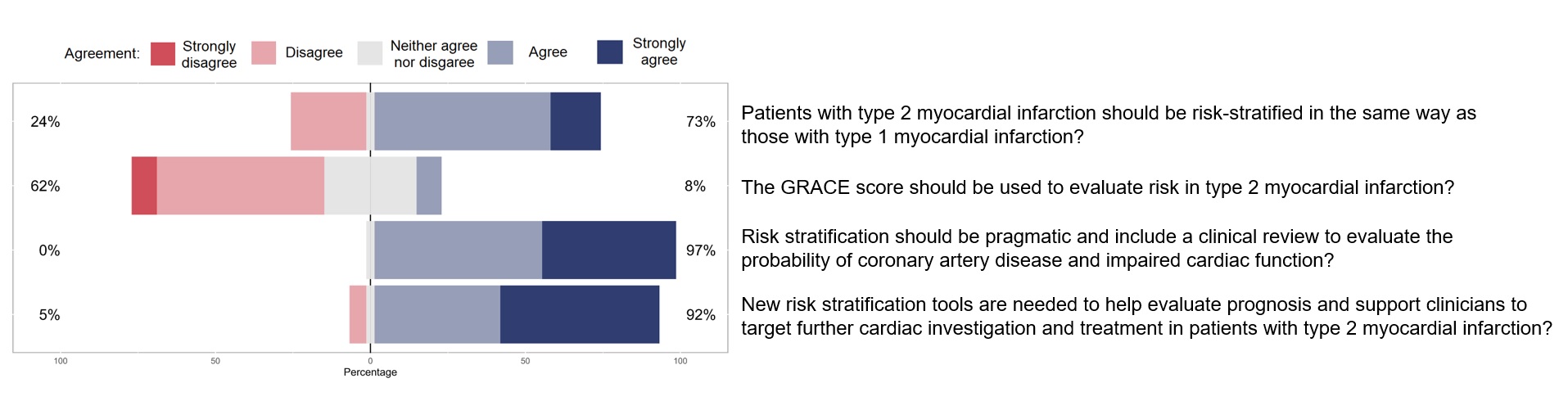
Supplementary Figure 3**: Statements and agreement on the risk-stratification of type 2 myocardial infarction.


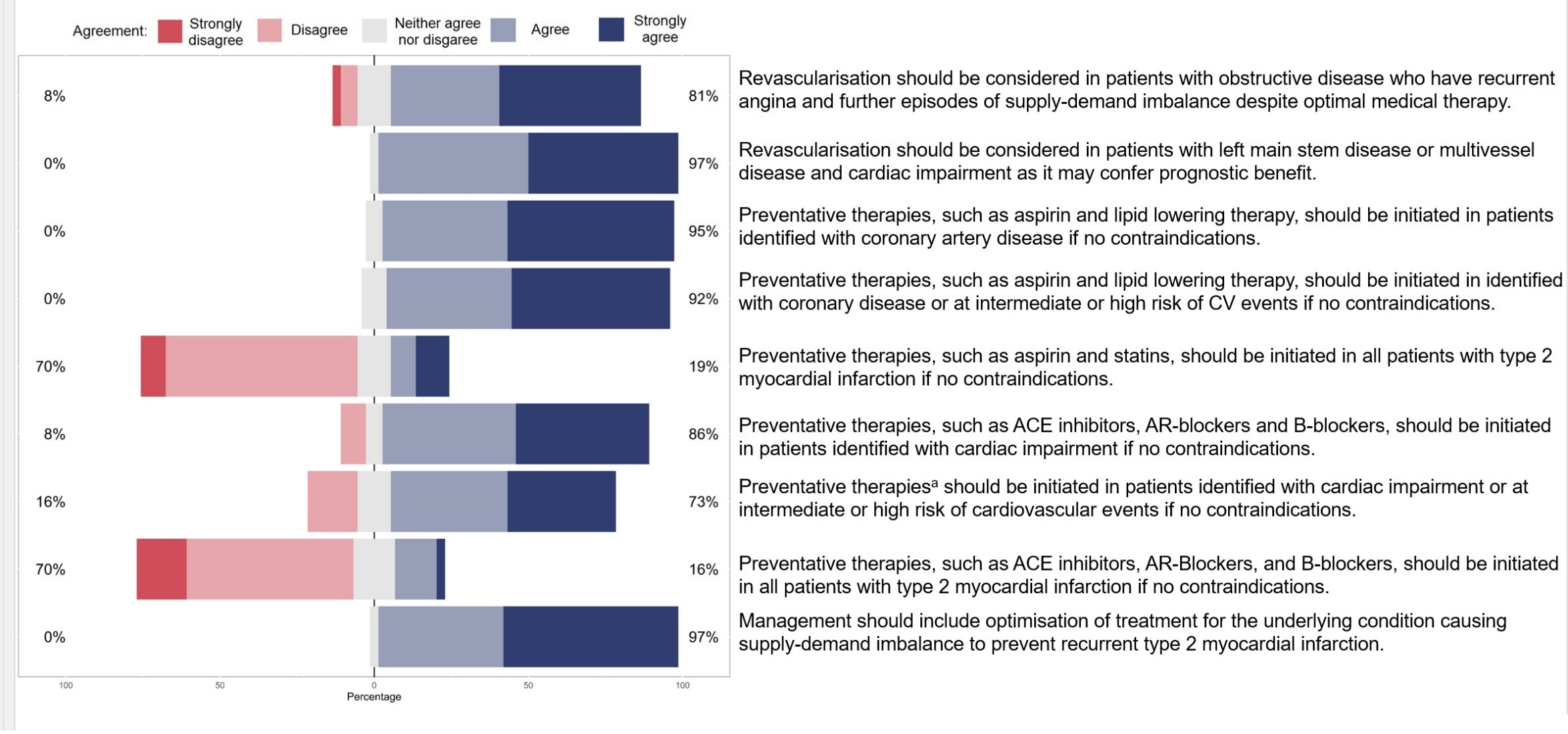
**Supplementary Figure 4:** Statements and agreement on the secondary prevention and revascularisation of management of type 2 myocardial infarction.

**
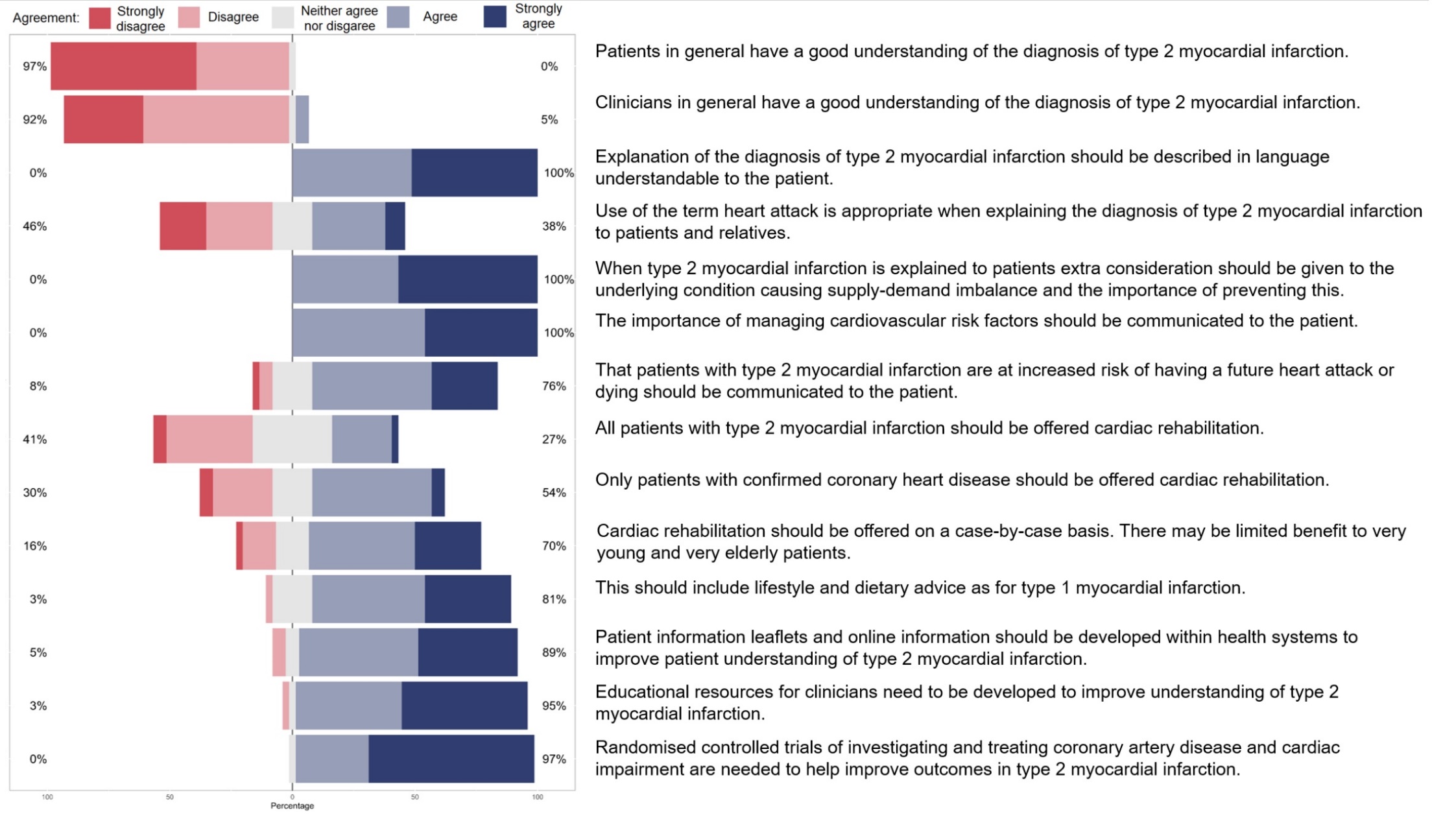
Supplementary Figure 5**: Statements and agreement on the communication and rehabilitation in patients with type 2 myocardial infarction.

**Supplementary Table 1.** Demographics of experts invited and participating in the Delphi study.

|  | **Invited** | **Participants** | |
| --- | --- | --- | --- |
|  |  | **Round 1** | **Round 2** |
| **Total** | **73** | **38** | **37** |
| **Sex (men)** | 63 (86%) | - | - |
| **Geographical location** |  |  |  |
| North America | 31 (42%) | - | - |
| Europe | 30 (41%) | - | - |
| Asia | 7 (10%) | - | - |
| Australia | 5 (7%) | - | - |
| **Clinical specialty** |  |  |  |
| Cardiology | 61 (84%) | 32 (84%) | 31 (84%) |
| Emergency medicine | 5 (7%) | 5 (13%) | 5 (14%) |
| Internal medicine | 4 (5%) | 1 (3%) | 1 (3%) |
| Clinical biochemistry | 2 (3%) | 0 (0%) | 0 (0%) |
| Other | 1 (1%) | 0 (0%) | 0 (0%) |

**Supplemental Table 2: Round two statements with number of participants in agreement with each statement and Likert scale criteria descriptive statistics.**

| **Domain and statement** | **Consensus** | | **Likert criteria** | | | |
| --- | --- | --- | --- | --- | --- | --- |
| **Definition of type 2 myocardial infarction** | **Strongly agree or agree (n)**  **(total = 37)** | **Percentage agreement** | **Median** | **Mean** | **IQR** | **SD** |
| The current definition of type 2 myocardial infarction should be reviewed | 29 | 78% | 2 | 1.97 | 1 | 1.09 |
| The diagnosis can be considered in patients with symptoms of myocardial ischemia even in the absence of objective evidence on the electrocardiogram or cardiac imaging | 27 | 73% | 2 | 2.46 | 2 | 1.28 |
| Objective evidence of myocardial ischaemia on the electrocardiogram or infarction on cardiac imaging should be a requirement for the diagnosis? | 15 | 41% | 4 | 2.86 | 3 | 1.36 |
| The diagnosis can be made only after exclusion of type 1 myocardial infarction | 17 | 46% | 3 | 2.89 | 2 | 1.20 |
| A cause of supply and demand imbalance should always be identified | 31 | 84% | 2 | 1.84 | 1 | 0.90 |
| Criteria should be defined that standardise the identification of myocardial oxygen supply demand imbalance for use in practice? | 14 | 38% | 4 | 3.27 | 2 | 1.37 |
| The above criteria should be used in practice* | 9 | 24% | 4 | 3.68 | 2 | 1.27 |
| Fixed criteria for myocardial oxygen supply-demand imbalance are not appropriate as they do not reflect individual susceptibility to myocardial ischemia? | 30 | 81% | 1 | 1.76 | 1 | 0.95 |
| Patients with any coronary mechanism of type 2 myocardial infarction should be classified as a subgroup of type 1 myocardial infarction | 21 | 57% | 2 | 2.68 | 3 | 1.60 |
| Patients with coronary embolism as the mechanism of type 2 myocardial infarction should be classified as a subgroup of type 1 myocardial infarction | 34 | 65% | 2 | 2.35 | 3 | 1.48 |
| Patients with coronary vasospasm as the mechanism of type 2 myocardial infarction should be classified as a subgroup of type 1 myocardial infarction | 23 | 62% | 2 | 2.49 | 3 | 1.48 |
| Patients with spontaneous coronary artery dissection as the mechanism of type 2 myocardial infarction should be classified as a subgroup of type 1 myocardial infarction | 26 | 70% | 2 | 2.24 | 2 | 1.42 |
| **Risk stratification in type 2 myocardial infarction** | |  |  |  |  |  |
| Patients with type 2 myocardial infarction should be risk-stratified in the same way as those with type 1 myocardial infarction | 27 | 73% | 2 | 2.35 | 1 | 1.03 |
| The GRACE score should be used to evaluate risk in type 2 myocardial infarction | 3 | 8% | 4 | 3.62 | 1 | 0.76 |
| Risk stratification should be pragmatic and include a clinical review to evaluate the probability of coronary artery disease and impaired cardiac function | 36 | 97% | 2 | 1.59 | 1 | 0.55 |
| New risk stratification tools are needed to help evaluate prognosis and support clinicians to target further cardiac investigation and treatment in patients with type 2 myocardial infarction | 34 | 92% | 1 | 1.62 | 1 | 0.79 |
| **Assessment for coronary artery disease in type 2 myocardial infarction** | |  |  |  |  |  |
| All patients with type 2 myocardial infarction should undergo investigation for the presence of coronary artery disease if not already known or contraindicated | 16 | 43% | 3 | 2.78 | 2 | 1.16 |
| Patients with type 2 myocardial infarction should undergo review and an assessment of the probability of coronary artery disease by a cardiologist to guide further investigation | 31 | 84% | 2 | 1.89 | 1 | 0.74 |
| In patients with a low probability or no cardiovascular risk factors investigation for coronary artery disease may not be required | 26 | 70% | 2 | 2.41 | 1 | 1.09 |
| In patients with low probability a functional assessment should be considered to assess for myocardial ischemia | 15 | 41% | 3 | 3.00 | 2 | 1.08 |
| In patients with intermediate probability of coronary artery disease computed tomography (CT) coronary angiography or a functional test should be considered | 35 | 95% | 2 | 1.81 | 0 | 0.52 |
| In patients with high probability, invasive coronary angiography is preferred to assess for coronary artery disease | 25 | 68% | 2 | 2.32 | 1 | 1.03 |
| In patients where there is uncertainty as to whether the presentation was due to atherothrombosis invasive coronary angiography should be performed | 32 | 86% | 2 | 1.86 | 1 | 0.79 |
| Functional or anatomical assessment for coronary artery disease should be delayed until the primary cause of supply-demand imbalance is resolved | 26 | 70% | 2 | 2.24 | 1 | 0.95 |
| In patients with a type 2 myocardial infarction a non-invasive approach should be considered first with a view to invasive imaging if needed | 24 | 59% | 2 | 2.41 | 1 | 0.98 |
| Exercise testing should be performed in patients with type 2 myocardial infarction | 4 | 11% | 4 | 3.49 | 1 | 0.90 |
| In patients with a high probability or ongoing myocardial ischaemia invasive coronary angiography should be performed during the inpatient stay | 33 | 89% | 1 | 1.62 | 1 | 0.76 |
| In patients with a low or intermediate probability of coronary artery disease and no ongoing myocardial ischemia further testing could be performed as an outpatient | 32 | 86% | 2 | 2.03 | 0 | 0.80 |
| **Assessment of cardiac function in type 2 myocardial infarction** | |  |  |  |  |  |
| All patients with type 2 myocardial infarction should undergo echocardiography to assess cardiac function | 26 | 70% | 2 | 2.08 | 2 | 1.23 |
| Selected patients with type 2 myocardial infarction should undergo echocardiography to assess cardiac function if evidence of significant myocardial ischemia or injury | 25 | 68% | 2 | 2.24 | 2 | 1.26 |
| Cardiac magnetic resonance imaging should be considered if no evidence of myocardial infarction is identified following echocardiography | 13 | 35% | 3 | 2.73 | 1 | 0.96 |
| Cardiac magnetic resonance imaging should be considered if the diagnosis remains uncertain following echocardiography | 25 | 68% | 2 | 2.22 | 1 | 0.85 |
| Assessment of cardiac function should be performed as an inpatient | 26 | 70% | 2 | 2.22 | 1 | 0.89 |
| Assessment of cardiac function should be performed as an outpatient | 4 | 11% | 4 | 3.59 | 1 | 0.90 |
| **Specialty management of type 2 myocardial infarction** | |  |  |  |  |  |
| Patients should be managed in a cardiac unit | 6 | 16% | 4 | 3.41 | 1 | 0.90 |
| Patients should be managed by a multi-disciplinary team with expertise for the range of conditions involved in their presentation | 30 | 81% | 2 | 2.03 | 0 | 0.76 |
| Patients should be managed by the specialty with expertise in the primary cause of supply-demand imbalance with guidance from cardiology | 31 | 84% | 2 | 1.97 | 1 | 0.87 |
| Patients should be reviewed by a cardiologist during their inpatient stay | 26 | 70% | 2 | 2.11 | 2 | 1.02 |
| Patients with ongoing myocardial ischemia should be reviewed urgently by a cardiologist | 34 | 92% | 1 | 1.57 | 1 | 0.87 |
| Cardiologists should be responsible for making the diagnosis, defining the aetiology and risk stratification in patients with type 2 myocardial infarction | 23 | 62% | 2 | 2.30 | 1 | 1.05 |
| Patients should be followed up in the cardiology outpatient clinic for assessment and optimisation of cardio-protective therapies | 11 | 30% | 3 | 2.81 | 1 | 0.91 |
| Only patients with a cardiac cause of supply-demand imbalance or found to have cardiac impairment or coronary artery disease should be followed up in the cardiology outpatient clinic | 25 | 68% | 2 | 2.43 | 2 | 1.14 |
| Patients should be followed up in the outpatient clinic of the specialty managing the primary cause of supply-demand imbalance | 32 | 86% | 2 | 2.00 | 0 | 0.62 |
| In patients with a poor prognosis from the primary cause of supply-demand imbalance outpatient follow up may not be practical or beneficial | 30 | 81% | 2 | 2.05 | 1 | 1.05 |
| **Secondary prevention and revascularisation in type 2 myocardial infarction** | |  |  |  |  |  |
| Revascularisation should be considered in patients with obstructive coronary artery disease who are likely to have recurrent symptoms of myocardial ischemia on effort or with further episodes of supply-demand imbalance despite optimal medical therapy | 30 | 81% | 2 | 1.84 | 1 | 1.01 |
| Revascularisation should be considered in patients with left main stem disease or multivessel disease and cardiac impairment as it may confer prognostic benefit | 36 | 97% | 2 | 1.54 | 1 | 0.56 |
| Preventative therapies, such as aspirin and lipid lowering therapy, should be initiated in patients identified with coronary artery disease if no contraindications | 35 | 95% | 1 | 1.51 | 1 | 0.61 |
| Preventative therapies, such as aspirin and lipid lowering therapy, should be initiated in identified with coronary artery disease or at intermediate or high risk of cardiovascular events if no contraindications | 34 | 92% | 1 | 1.57 | 1 | 0.65 |
| Preventative therapies, such as aspirin and statins, should be initiated in all patients with type 2 myocardial infarction if no contraindications | 7† | 19% | 4 | 3.49 | 1 | 1.12 |
| Preventative therapies, such as ACE inhibitors, angiotensin receptor blockers, and beta-blockers, should be initiated in patients identified with cardiac impairment if no contraindications | 36 | 86% | 2 | 1.78 | 1 | 0.89 |
| Preventative therapies, such as ACE inhibitors, angiotensin receptor blockers, and beta-blockers, should be initiated in patients identified with cardiac impairment or at intermediate or high risk of cardiovascular events if no contraindications | 27 | 73% | 2 | 2.08 | 2 | 1.06 |
| Preventative therapies, such as ACE inhibitors, angiotensin receptor blockers, and beta-blockers, should be initiated in all patients with type 2 myocardial infarction if no contraindications | 6^†^ | 16% | 4 | 3.68 | 1 | 1.00 |
| Management should include optimisation of treatment for the underlying condition causing supply-demand imbalance to prevent recurrent type 2 myocardial infarction | 36 | 97% | 1 | 1.46 | 1 | 0.56 |
| **Communication** |  |  |  |  |  |  |
| Patients in general have a good understanding of the diagnosis of type 2 myocardial infarction | 0^†^ | 0% | 5 | 4.57 | 1 | 0.55 |
| Clinicians in general have a good understanding of the diagnosis of type 2 myocardial infarction | 2^†^ | 5% | 4 | 4.18 | 1 | 0.74 |
| Explanation of the diagnosis of type 2 myocardial infarction should be described in language understandable to the patient | 37 | 100% | 1 | 1.47 | 1 | 0.51 |
| Use of the term heart attack is appropriate when explaining the diagnosis of type 2 myocardial infarction to patients and relatives | 14 | 38% | 3 | 3.18 | 2 | 1.29 |
| When type 2 myocardial infarction is explained to patients extra consideration should be given to the underlying condition causing supply-demand imbalance and the importance of preventing this | 37 | 100% | 1 | 1.42 | 1 | 0.50 |
| The importance of managing cardiovascular risk factors should be communicated to the patient | 37 | 100% | 2 | 1.55 | 1 | 0.51 |
| That patients with type 2 myocardial infarction are at increased risk of having a future heart attack or dying should be communicated to the patient | 28 | 76% | 2 | 2.08 | 1 | 0.95 |
| All patients with type 2 myocardial infarction should be offered cardiac rehabilitation? | 10 | 27% | 3 | 3.16 | 2 | 0.96 |
| Only patients with confirmed coronary heart disease should be offered cardiac rehabilitation | 20 | 54% | 2 | 2.74 | 2 | 1.06 |
| Cardiac rehabilitation should be offered on a case-by-case basis. There may be limited benefit to very young and very elderly patients | 26 | 70% | 2 | 2.21 | 2 | 1.08 |
| This should include lifestyle and dietary advice as for type 1 myocardial infarction? | 30 | 81% | 2 | 1.87 | 1 | 0.79 |
| Patient information leaflets and online information should be developed within health systems to improve patient understanding of type 2 myocardial infarction? | 33 | 89% | 2 | 1.76 | 1 | 0.80 |
| Educational resources for clinicians need to be developed to improve understanding of type 2 myocardial infarction | 35 | 95% | 1 | 1.55 | 1 | 0.69 |
| Randomised controlled trials of investigating and treating coronary artery disease and cardiac impairment are needed to help improve outcomes in type 2 myocardial infarction | 36 | 97% | 1 | 1.34 | 1 | 0.54 |

†Consensus gained on disagreement *Criteria defined by Saaby et al 2014

**Supplementary Table 3: Round three statements with number of participants in agreement with each statement and Likert scale criteria descriptive statistics.**

|  | **Consensus** | | **Likert criteria** | | | |
| --- | --- | --- | --- | --- | --- | --- |
| **Statement** | **Strongly agree or agree (n)**  **(total = 49)** | **Percentage agreement** | **Median** | **Mean** | **IQR** | **SD** |
| Objective evidence of myocardial ischaemia on the electrocardiogram should be a requirement for the diagnosis | 21 | 43% | 3 | 2.90 | 2 | 1.28 |
| Objective evidence of new infarction on echocardiography or cardiac magnetic resonance imaging should be a requirement for the diagnosis | 19 | 39% | 3 | 2.98 | 2 | 1.23 |
| Patients with any pathology leading to partial or complete coronary occlusion (atherothrombosis, dissection, embolism, and vasospasm) should be classified as a subgroup of type 1 myocardial infarction | 28 | 57% | 2 | 2.59 | 3 | 1.53 |
| The diagnosis of type 2 myocardial infarction should be limited to those with a presumed or demonstrable coronary cause (dissection, embolism, vasospasm, or fixed atherosclerosis with supply-demand imbalance) | 19 | 39% | 4 | 3.22 | 2 | 1.33 |
| The diagnosis of type 2 myocardial infarction should be further subclassified into those with (type 2 a) or without (type 2b) a presumed or demonstrable coronary cause (dissection, embolism, vasospasm, or fixed atherosclerosis with supply demand imbalance) | 31 | 63% | 2 | 2.49 | 2 | 1.26 |
| In patients with a high probability of coronary artery disease in whom further investigation is appropriate, invasive coronary angiography is preferred | 26 | 73% | 2 | 2.24 | 2 | 1.15 |
| Cardiac magnetic resonance imaging should be considered if the diagnosis remains uncertain following echocardiography and/or coronary angiography | 33 | 88% | 2 | 1.88 | 1 | 0.73 |
| Patients with a cardiac cause of supply-demand imbalance or found to have cardiac impairment or coronary artery disease should be followed up in the cardiology outpatient clinic | 48 | 98% | 2 | 1.65 | 1 | 0.60 |
| Use of the term heart attack is appropriate when explaining the diagnosis of type 2 myocardial infarction to patients and relatives | 23 | 47% | 3 | 2.82 | 2 | 1.20 |
| Use of the term heart attack is appropriate when explaining the diagnosis of type 2 myocardial infarction to patients and relatives where infarction was caused by a coronary mechanism | 43 | 88% | 2 | 1.86 | 1 | 0.74 |
| Use of the term heart attack is appropriate when explaining the diagnosis of type 2 myocardial infarction to patients and relatives where there is new evidence of myocardial infarction on cardiac imaging | 41 | 84% | 2 | 2.02 | 0 | 0.88 |

**Appendix**

**Quotes from free text in round 2 from panel**

- “I think that we must offer some broad guidelines but not require very specific recommendations until further clinical trials concerning NSTEMI type 2 have been completed.”
- “I think there is significant overlap between type 2 MI as proposed and ischaemic myocardial injury that is multifactorial. A diagnostic label is useless without a treatment strategy, and this should be the aim.”
- “Ischemic threshold a key concept.”
- “There should always be a caveat to only target risk stratification to patients who will have a chance to benefit from intervention.”
- “The challenge here is that not only is it important to risk stratify but also try to disentangle cardiovascular risk from competing risk of non-cardiovascular disease. Perhaps we need risk tools to predict non-CV mortality and separate risk tool for MACE (CV death and MI).”
- “To detect a non-atherothrombotic coronary mechanism (thus a type-2 MI) you often need an invasive coronary angiography.”
- “Regarding the above, we should be factoring in life expectancy into these decisions before saying “routine evaluation” is needed. If someone has a life-expectancy of <1 year I wouldn’t do any testing for coronary disease.”
- “I think that troponin levels should be considered in this discussion. Patients with a minimal troponin rise and or fall and having a type 2 NSTEMI probably have quite different underlying pathophysiology than ones with higher levels.”
- “Follow-up will depend on the work-up done while in-hospital. Perhaps more reasonable to frame as outpatient cardiology follow-up is warranted in those with T2MI and evidence of CAD or structural heart disease or those who did not undergo any inpatient cardiac evaluation provided appropriate in the context of potential competing risks from non-cardiac medical conditions.”
- “one size does not fit all.”
- “TTE is really necessary in all type 2 MI patients, to evaluate for regional wall motion abnormalities, reduced LV ejection fraction, infiltrative myocardial disease, valvular heart disease, pericardial pathologies, etc.”
  CMR should be used selectively, perhaps most beneficial in those with large amounts of myocardial injury, in whom the diagnosis is uncertain.
- “Agree with most of this with the exception of the statements that ‘all’ patients and ‘cardiologist’. These are overly restrictive and largely health system dependent. Definition should focus on the diagnostic threshold/approach”.
- “Patients with type 2 MI are usually frailer and invasive angiography may not be best approach if non-invasive strategy available. Also exercise testing has same challenges”.
- “iSGLT2 could be considered in some patients”.
- “When hypotension is the cause of type 2 MI, ACE inhibitors, ARBs, and BB may not be prudent. When bleeding is the cause of type 2 MI, aspirin/anti-thrombotic may not be prudent. Although it needs to be confirmed in a RCT, I think that medial therapy for type 2 MI is essential.”
- “Difficult to tell patients they are at increased risk of heart attack when we have no evidence to guide investigation or treatment which modifies outcomes.”
- “Cardiac rehab is generally good but is a limited resource and should be used in patients most likely to derive benefit”.
- “need to further define groups involved prior to trials.”

**Appendix**

**Round one questions to potential collaborators.**

1. Please indicate whether you are a practicing clinician and your area of practice (e.g. cardiology, emergency medicine, internal medicine).

2. Can you give a brief description of the diagnostic criteria for type 2 myocardial infarction that you use in clinical practice?

3. What is your understanding of acute myocardial injury? Do you manage these patients differently from those with type 2 myocardial infarction? if so, how?

4. In your institution which specialty is predominantly responsible for the inpatient management of patients with type 2 myocardial infarction? Do you feel this is appropriate?

5. Are all or most patients assessed by a cardiologist during their inpatient stay? Do you feel this is appropriate and if not, what are the barriers?

6. Following discharge are patients routinely followed up in the outpatient clinic? If so, by which specialty is follow up arranged and do you feel this is best practice?

7. Do you routinely assess the probability of coronary or structural heart disease in patients with type 2 myocardial infarction? If so, how?

8. Do you routinely use scores to assess risk of coronary heart disease or prognosis in patients with type 2 myocardial infarction in your practice? Do you feel this would be appropriate?

9. We know that type 2 myocardial infarction can occur in a wide range of clinical conditions and that outcomes are often poor. Do you feel there are any subgroups of patients with type 2 myocardial infarction that may be more likely to benefit from further investigation or treatment? Or on the contrary, are there any that are at increased risk of iatrogenic harm?

10. Do you routinely recommend investigations for coronary heart disease in patients with type 2 myocardial infarction? If not, are there selected patients in whom you would recommend coronary investigation and how do you identify these patients? Do you prefer non-invasive or invasive coronary investigation and why? When would you schedule coronary investigation?

11. Do you routinely recommend imaging to evaluate cardiac function in patients with type 2 myocardial infarction? If not, are there selected patients in whom you would recommend imaging and how do you identify these patients? What imaging methods would you consider and what is your preferred approach? When would you schedule imaging to assess cardiac function?

12. In patients with type 2 myocardial infarction found to have obstructive coronary artery disease would you routinely recommend revascularisation? If not, on what basis would you recommend revascularisation?

13. In patients with type 2 myocardial infarction are there any secondary prevention measures or medication you would consider to mitigate future cardiovascular risk? If so, on what basis do you recommend these measures?

14.  In your practice, how do you explain what a type 2 myocardial infarction is to your patient, and do you feel that most have a good understanding of this condition?

15. Are patients routinely offered the opportunity to attend cardiac rehabilitation in your institution and if not do you feel this would be beneficial?

16. Please identify any other considerations which you consider good practice in the diagnosis, assessment, investigation, treatment and communication with patients who have type 2 myocardial infarction.
